# Allergy—A New Role for T Cell Superantigens of *Staphylococcus aureus*?

**DOI:** 10.3390/toxins12030176

**Published:** 2020-03-12

**Authors:** Goran Abdurrahman, Frieder Schmiedeke, Claus Bachert, Barbara M. Bröker, Silva Holtfreter

**Affiliations:** 1 Department of Immunology, University Medicine Greifswald, 17475 Greifswald, Germany; goran.abdurrahman@uni-greifswald.de (G.A.); frieder.schmiedeke@uni-greifswald.de (F.S.); broeker@uni-greifswald.de (B.M.B.); 2 Upper Airways Research Laboratory, Department of Otorhinolaryngology, Ghent University, 9000 Ghent, Belgium; Claus.Bachert@UGent.be; 3 Current address: Department of Medicine Solna, Immunology and Allergy Research Unit, Karolinska Institute, 171 77 Stockholm, Sweden

**Keywords:** *Staphylococcus aureus*, superantigens, T cells, allergy, sensitization, IgE, T cell superallergen

## Abstract

*Staphylococcus aureus* superantigens (SAgs) are among the most potent T cell mitogens known. They stimulate large fractions of T cells by cross-linking their T cell receptor with major histocompatibility complex class-II molecules on antigen presenting cells, resulting in T cell proliferation and massive cytokine release. To date, 26 different SAgs have been described in the species *S. aureus*; they comprise the toxic shock syndrome toxin (TSST-1), as well as 25 staphylococcal enterotoxins (SEs) or enterotoxin-like proteins (SEls). SAgs can cause staphylococcal food poisoning and toxic shock syndrome and contribute to the clinical symptoms of staphylococcal infection. In addition, there is growing evidence that SAgs are involved in allergic diseases. This review provides an overview on recent epidemiological data on the involvement of *S. aureus* SAgs and anti-SAg-IgE in allergy, demonstrating that being sensitized to SEs—in contrast to inhalant allergens—is associated with a severe disease course in patients with chronic airway inflammation. The mechanisms by which SAgs trigger or amplify allergic immune responses, however, are not yet fully understood. Here, we discuss known and hypothetical pathways by which SAgs can drive an atopic disease.

## 1. Introduction

*Staphylococcus (S.) aureus* is a multifaceted human pathobiont. The most frequent encounter with *S.* aureus is symptom-free colonization, with 20% of the human population being persistently colonized, and the remainder being intermittently colonized [1,2]. Moreover, these bacteria cause a wide spectrum of illnesses, ranging from self-limiting food poisoning and skin and soft tissue infections to life-threatening diseases, such as pneumonia, endocarditis, and sepsis [3]. In addition, more recent evidence suggests an unexpected role of *S. aureus* in allergic diseases [4].

The capability of *S. aureus* to cause such a broad range of clinical outcomes is based on an abundance of adhesins, exoenzymes, immune evasion factors, and virulence factors, which facilitate attachment, colonization, tissue invasion, toxinosis, immune evasion, and allergic reactions [5]. Superantigens (SAgs) are the most notorious of this large arsenal of staphylococcal virulence factors. These exotoxins activate large subpopulations of T lymphocytes, causing a massive cytokine release which may lead to systemic shock. On top, there is accumulating evidence for a role of SAgs in triggering and amplifying allergic responses [6].

This review:

(1) Provides an overview on the function and diversity of staphylococcal superantigens (SAgs),
(2) Reports on advances in the development of SAg vaccines,
(3) Summarizes recent epidemiological data on the involvement of SAgs in allergy,
(4) Outlines mechanisms by which SAgs could induce or amplify allergic responses,
(5) Elaborates on the evolutionary advantage gained by the production of SAgs, and finally,
(6) Discusses knowledge gaps that should be addressed in future research.

### 1.1. SAgs are Extremely Potent T Cell Mitogens

SAgs are the most potent T cell mitogens known. Low picomolar and even femtomolar concentrations are sufficient to trigger oligoclonal T cell activation, resulting in an immense cytokine release [6]. Hence, the term “superantigen” seems appropriate [7,8]. In contrast, a B cell SAg, e.g., the staphylococcal protein A, binds to the B cell receptor and induces polyclonal B cell activation [9]. SAgs have evolved in parallel not only in different bacteria but also in viruses; the most famous are the phylogenetically related enterotoxins secreted by *S. aureus* and *Streptococcus pyogenes* [10].

**Figure 1:**
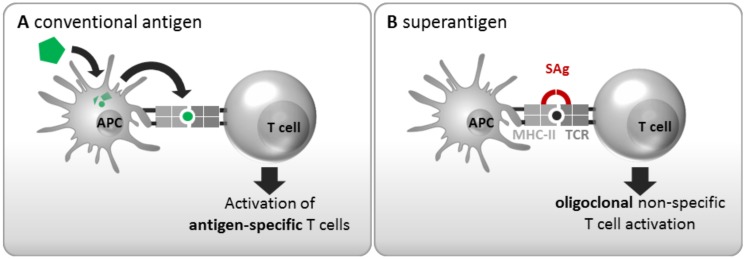
SAgs induce oligoclonal T cell activation by circumventing conventional antigen presentation pathways. (**A**) Upon uptake, conventional antigens are processed into short peptides and presented on MHC-II molecules to CD4^+^ T cells. Only those rare T cells with the complementary TCR specificity will be activated (one out of 10^4^–10^5^). (**B**) In contrast, SAgs circumvent this highly specific interaction by directly cross-linking TCRs and MHC-II molecules outside their peptide binding sites, resulting in oligoclonal T cell activation. MHC-II: Major histocompatibility complex class-II, TCR: T cell receptor, SAg: Superantigen, APC: Antigen presenting cell. Arrows indicate the sequence of events.

The molecular mechanism underlying oligoclonal T cell stimulation by SAgs have been resolved in the past decades and are elaborated below (Section 3.2). Briefly, SAgs act by circumventing the physiological antigen processing and presentation pathways. Conventional antigens are engulfed and processed by antigen presenting cells (APCs, e.g., dendritic cells, B cells, and macrophages). The generated antigenic peptides are presented on major histocompatibility complex class II (MHC-II) molecules to CD4^+^ T cells, which discern the complex via the hypervariable loops of their T cell receptor (TCR) α and β chains. Only Th cells with complementary receptor specificity are activated, resulting in clonal expansion, cytokine secretion, and B cell help (Figure 1A). SAgs can short-circuit this highly specific interaction between APCs and T cells by binding both TCRs and MHC-II molecules outside of their peptide binding sites (Figure 1B). Hence, T cells are triggered independently of their antigen specificity, eventually leading to an activation of up to 20% of all T cells. Activated T cells will strongly proliferate and release large amounts of cytokines, predominantly interleukin (IL)-2, tumour necrosis factor α (TNF-α), and interferon γ (IFN-γ) [11,12,13]. This proliferative stage can be followed by a profound state of T cell exhaustion, i.e., unresponsiveness, or even cell death [13]. On the APC side, SAg-induced activation can have various outcomes depending on the cell type. In the case of monocytes for instance, activation is triggered by dimerization of MHC-II molecules and/or signaling via CD40 leading to the secretion of TNF-α, IL-1β, and IL-6 [11,14,15,16]. SAgs have also been shown to inhibit monocyte proliferation [16].

### 1.2. Staphylococcal SAgs are Highly Diverse

To date, 26 different SAgs have been described in the species *S. aureus*. They comprise the toxic shock syndrome toxin (TSST-1), 11 staphylococcal enterotoxins (SEA–SEE, SEG–SEI, SER–SET), as well as 14 SE-like proteins (SElJ–SElQ, SElU–SElZ). While SEs are toxins with demonstrated emetic activity, the SEl proteins are not emetic in a primate model or have yet to be tested.

Most SAg genes are encoded on mobile genetic elements (MGEs), such as phages, pathogenicity islands, and plasmids, which can be exchanged between *S. aureus* isolates by horizontal gene transfer [11]. In contrast, the enterotoxin gene cluster, *egc*, including *seg*, *sei*, *sem*, *sen*, *seo*, and sometimes *seu*, is located on the genomic island vSAβ, which cannot be mobilized [17]. Moreover, some core genome-encoded SAgs exist that are present in all *S. aureus* isolates, i.e., SElX, SElY, and SElZ.

Due to their locations on MGEs and the vSAβ island, the SAg gene repertoire of clinical *S. aureus* isolates is highly diverse, and even closely related isolates can differ in their SAg gene patterns [18]. The distribution of the SAg-carrying MGEs across the different *S. aureus* lineages is not random. In fact, each lineage is characterized by a more or less restricted SAg gene pattern. For instance, the vSAβ-encoded *egc* SAgs are strictly linked to the clonal background. Moreover, the transfer of MGE-encoded SAgs is limited to certain lineages due to lineage-specific restriction/modification systems. Both mechanisms contribute to lineage-specific rather than random SAg repertoires.

SAg gene expression is tightly regulated. While most SAg genes are transcribed in the stationary growth phase in vitro, *egc* SAgs are expressed at low bacterial densities [17,19,20]. This differential regulation might explain why humans rarely harbor antibodies against these SAgs, while serum immunoglobulin (Ig) G antibodies against non-*egc* SAgs are very common, even in the healthy population [19,21]. Which SAg genes are expressed under atopic conditions in vivo is still largely unknown. However, the available data suggest that SAg genes may be expressed during symptom-free colonization as well as under atopic conditions. For instance, our group determined the expression profiles of three SAgs in *S. aureus* directly isolated from the nose of healthy persistent carriers and observed that *sea*, *sec*, and the *egc* SAg *selo* were all expressed during nasal colonization [22]. Similarly, *tst* transcripts were detected in the nose of a healthy carrier by RNAseq analysis [23]. In addition, SAgs were detected within nasal polyp tissue in patients with chronic rhinosinusitis (CRS) [24,25]. More recently, more than 600 proteins released by *S. aureus* were identified by high resolution mass spectrometry in the upper airway of patients with CRS; among these were also SEs [26]. Finally, SEA was expressed by *S. aureus* Newman cultivated in lung surfactant [27]. Overall, these studies suggest that SAg genes are indeed expressed under atopic conditions in vivo.

The 26 known staphylococcal SAgs share between 15.5% to 90% sequence homology on the protein level [28]. Despite this variable degree of homology, all SAgs share a similar three-dimensional structure, consisting of two globular domains, and the same binding partners [6,29,30]. Recent structural studies, however, have revealed that SAgs are able to crosslink MHC-II molecules and TCRs in a variety of ways [31]. This pronounced diversity of interactions hampers the development of a universal SAg vaccine. Only SAg vaccines that target several specific binding domains will be able to induce cross-protection against many SAgs.

Vaccine development against staphylococcal SAgs is also hindered by another property: Due to their highly specific interaction with conserved regions of MHC alleles and TCR variants, these toxins act to some degree host specific. For instance, compared to human T cells, the SAg’s mitogenic activity is reduced by a factor of 100–1000 in murine and rat-derived T cells [11,19,32,33]. The only known susceptible animal species to develop human-like enterotoxigenic disease upon SAg exposure are non-human primates [30]. This host-specific activity limits the range of animal models suitable for studying SAg vaccines.

### 1.3. SAgs Can Induce Various Clinical Pictures

SAgs can trigger a range of clinical pictures, including toxinoses. The most frequent SAg-induced toxinosis is staphylococcal food poisoning, characterized by nausea and violent vomiting, which is usually self-limiting [30]. SAgs are highly stable molecules, resistant to heat, low pH, and digestion by pepsin and trypsin [33]. Hence, SAgs produced by staphylococci on raw food are able to endure the cooking process and transit the acidic stomach without damage. Upon entering the gut, SAgs probably bind to a yet unidentified receptor on the surface of the submucosal mast cells and induce 5-HT release. 5-HT subsequently depolarizes the vagal afferent nerves resulting in stimulation of the brain stem emetic loci to initiate the vomiting reflex [30].

Once they enter circulation in sufficient amounts, SAgs can trigger the toxic shock syndrome (TSS). TSS is an acute and potentially fatal illness characterized by a high fever, diffuse erythematous rash, hypotension, involvement of three or more organ systems, as well as desquamation of the skin one to two weeks after onset (if not fatal before this time) [34]. This rare toxinosis involves the release and systemic spread of SAgs from local infection sites, mostly from the vagina as a result of tampon misusage, but also from infected wounds [34]. After entering the circulation, these toxins trigger massive systemic T cell activation and cytokine release, leading to fever, inflammation, vascular leakage, hypotension, multiorgan injury, and sometimes eventually death. TSS is a rare disease as most humans have high titers of neutralizing serum antibodies [35,36,37].

## 2. Vaccination against SAgs

SAgs are of interest as vaccine candidates due to their implications in bacterial pathogenesis and the lack of causal treatment strategies for SAg-induced diseases. However, due to their extremely high mitogenic activity, only fragments or toxoids, i.e., SAg mutants lacking the ability to cross-link TCR and MHC-II, can be used for vaccination. The structure–function relationship of several SAgs has been resolved in the past 25 years [6,38,39,40,41,42,43,44]. On that basis, inactivating mutations have been predicted and experimentally confirmed for a number of staphylococcal SAgs [45,46,47]. The development of detoxified SEB and TSST-1 mutants is most advanced [48,49]. An effective vaccine against SAgs would encompass the detoxified antigen in combination with adjuvants that stimulate a robust antibody response necessary for direct neutralization of the toxin and an appropriate T cell response targeted at clearing the pathogen [50].

Early vaccination studies with SAgs in animal models showed promising results in terms of protection against SAg-induced diseases. For instance, mice vaccinated with a SEA mutant with strongly reduced MHC-II binding and diminished mitogenic activity (SEA Y92A) were protected from subsequent lethal SEA-induced toxic shock [51]. Similarly, another SEA mutant (SEA D227A) devoid of toxic properties induced neutralizing antibodies and provided protection against SEA-induced emesis in house musk shrews [52]. There is also evidence that SAg vaccines can protect against *S. aureus* infections. Several studies demonstrated that vaccination with SAg toxoids provided protection from subsequent *S. aureus* sepsis in mice and rabbits [53,54,55,56].

The protective immunity induced by vaccination with SAg toxoids seems to depend on both anti-SAg antibodies and T cells. Indeed, there is strong evidence for a protective role of neutralizing anti-SAg antibodies: While more than 90% of healthy adults have high anti-TSST-1 antibody titers, these are absent in 90% of patients with menstruation-associated TSS [34,37]. Similarly, transfer of serum from SAg-vaccinated mice to naive mice conferred partial protection from *S. aureus* infection [53,57]. T cells also contribute to protection via IL-17-dependent mechanisms [58]. Therefore, an effective SAg toxoid vaccine would elicit both neutralizing anti-SAg antibodies and protective SAg-specific T cell responses. Antibodies are required to neutralize the toxicity of SAgs and dampen the inflammatory response early on during an infection while allowing Th17 cells to take action and clear the infection [47]. Moreover, any SAg toxoid vaccines should be adjuvanted and/or administered in a way that induces a protective Th1/Th17 response while preventing unwanted Th2/Treg responses.

The high variability of SAgs on the protein level, however, impedes vaccine development against SAgs [28]. There is evidence from both animal experiments and human studies that the adaptive immune system meticulously differentiates between the numerous SAg proteins [35,59,60,61,62]. For instance, SAg-vaccinated mice mount an antibody response that is specific for the vaccine SAg but is 10 times less effective against other SAgs [61]. Similarly, *S.* *aureus* carriers harbor highly specific anti-SAg antibodies that neutralize the SAgs of their colonizing strains, but are less (or not at all) effective against other SAgs [35]. Thus, vaccinating with a single SAg toxoid is probably not sufficient for effective protection against all SAgs. A way out could be targeting the immune response to conserved residues, for instance by a synthetic SAg vaccine. In fact, a synthetic SAg vaccine based on two conserved regions within the staphylococcal SAgs induced antibodies that neutralized the mitogenic activity of five tested *S. aureus* SAgs in vitro. Interestingly, the synthetic SAg peptide also acts as an inhibitor by binding tightly to the MHC-II molecule. Application of the peptide 2 and 1 h before SAg challenge rescued the mice from SEB- and TSST-1-induced lethality [63]. Recently, administration of a fusion protein (TBA_225_) consisting of three toxoids, TSST-1, SEB, and SEA, protected mice against the challenge with any of these three SAgs in a murine toxic shock model [64]. Polyclonal antibodies raised in rabbits against this vaccine efficiently neutralized the superantigenicity of the vaccine SAgs and showed strong cross-reactivity to SEC and SEH. However, their ability to neutralize other SAgs, including SED, SEE, and SEK, was reduced by a factor of 10–100 [64]. To conclude, despite promising recent advances in preclinical vaccine research there is still some work to be done to reach the grail of a cross-neutralizing vaccine against all *S. aureus* SAgs.

To date, a TSST-1 toxoid (double mutant G31R-H135A) and an SEB toxoid (triple mutant L45R-Y89A-Y94A), both supplemented with aluminum hydroxide adjuvant, have been tested in phase 1 clinical trials. Both studies showed the vaccines to be safe and well tolerated among healthy vaccines (Table 1) [48,49]. Moreover, both toxoids were still immunogenic and induced a neutralizing antibody response. These studies represent an important step in the development of SAg vaccines. However, since *S. aureus* produces a plethora of toxins apart from SAgs, a vaccine targeting only SAgs might not provide broad protection against a challenging pathogen like *S. aureus*. Hence, vaccination studies should include SAgs along with other virulence factors in a multivalent vaccine [47].

## 3. SAgs and Allergy

### 3.1. Epidemiological Evidence for SAg Involvement in Allergy

*S. aureus* can frequently be found in the nasal passages and on the skin of healthy people, but is reported to be even more frequent on the mucosal and skin surfaces in allergic rhinitis, asthma, CRS, and atopic dermatitis patients [65]. While approximately 20% of the healthy population are persistent carriers of *S. aureus* [1], patients with airway diseases show significantly higher rates of *S. aureus* colonization with up to 90% in patients with nasal polyps, with the highest prevalence in patients with comorbid asthma and aspirin sensitivity [66,67]. Furthermore, in the upper airways, *S. aureus* may grow intramucosally and even reside intracellularly [68,69]. *S. aureus* cells within nasal polyp tissue release numerous toxins and immune evasion molecules into the local environment, including SAgs but also staphylococcal serine protease-like (Spl) proteins, which are also suspected to play a role in allergies [26,70,71]. When the bacterium comes in contact with the immune system, specific IgG antibodies are formed against different bacterial components. However, in a type 2-biased local immune reaction, SEs, but also Spls and other immune active proteins may also give rise to the formation of IgE antibodies [70,72]. In particular, the presence of SE-IgE has been associated with the severity of any of the mentioned airway and skin diseases [73,74].

The presence of *S. aureus* and its allergenic components in home dust extracts has broadened the range of possible sources for contact with these bacteria. The SAgs SEA, SEB, and SEC were found in 36–60% of the house dust samples [75]. House dust mites, that live in close association with humans, obviously act as carriers for *S. aureus* and thus IgE-reactive bacterial antigens [76]. Moreover, *sea*-*sed* genes were detected in bedroom dust in 21% to 63% of samples [77].

There is accumulating evidence today that SE-IgE is present in a relevant subgroup of patients suffering from upper and lower type 2 airway diseases [4,78,79]. In a multicenter European study, 2908 subjects representative of the general population answered questionnaires on their disease, underwent skin prick tests (SPT) for common aeroallergens, and provided blood for measurement of total serum IgE and serum SE-IgE, using a mix of three staphylococcal superantigens (SEA, SEC, and TSST-1) [80]. From the results, 29.3% of the population was positive for SE-IgE with significant geographic variation. SE-IgE was more common in smokers with ≥15 pack years (OR 1.70, 95% confidence interval (CI) 1.34–2.60 *p* < 0.001). Moreover, SE-IgE was associated with asthma (OR 2.10, 95% CI 1.60–2.76, *p* = 0.001) in a serum concentration-dependent manner (OR 1.20, 1.74, 2.57 for the first, second, and third tertile, respectively, above 0.10 kUA/L; kilounits of allergen-specific IgE per liter) independent of the SPT results for inhalant allergens. Total IgE concentrations were higher in those with positive SE-IgE than in those with positive SPT. This was the first study to show that SE-IgE is significantly and independently associated with asthma in the general population.

In a smaller asthma cohort, SE-IgE positivity in serum was significantly more frequent in patients with severe asthma than in healthy control subjects (60 vs. 13%, *p* < 0.001) [67]. Logistic regression analyses demonstrated significantly increased risks for SE-IgE positive subjects compared to negative subjects to have any asthma (OR 7.25, 95% CI 2.7–19.1; *p* < 0.001) or severe asthma (OR 11.09, 95% CI 4.1–29.6; *p* < 0.001) [67], whereas grass pollen- or house dust mite-specific IgE was not associated with any risk. SE-IgE in asthmatics was associated with significantly increased oral steroid use, more frequent hospitalizations within the last year, and a lower lung function (FEV1% predicted value). SE-IgE can be found in atopic and non-atopic patients; in non-atopic asthma, total serum IgE levels were significantly increased in patients sensitized to SEs compared to patients non-sensitized to SEs. Out of 224 patients with non-atopic and/or late-onset asthma, 47% patients were sensitized to SEs [81]. The study concluded that SE sensitization may contribute to Th2-mediated inflammation in non-atopic patients.

Song et al. reported similar observations from Korea. Serum SE-IgE concentrations were significantly higher in asthmatics than controls [66]. Elderly asthma patients with high SE-IgE levels had more severe asthma, sputum eosinophilia, and CRS compared to those with lower SE-IgE levels. The IDENTIFY study was conducted in Germany and focused on severe asthmatics previously considered to be non-atopic based on a standard SPT [82]. In the non-atopic asthma group (*n* = 188), allergic sensitization to at least one allergen was additionally detected in 52% of patients, with the most frequent sensitizations to the SAgs SEB (app. 25%) and SEA (app. 15%); 8% of the patients were mono-sensitized to SEA or SEB. This still may be an underestimation of sensitizations to *S. aureus* SAgs, as the species harbors 26 SAg genes, of which only two have been tested.

In a nested case-control study using the 20-year Epidemiological study on the Genetics and Environment of Asthma (EGEA) cohort, including 225 adults, SE sensitization varied between 39% in controls to 58% and 76% in mild and severe asthma, respectively [83]. SE sensitization was associated with an increased risk for severe asthma (adjusted OR 2.69, 95% CI 1.18–6.15) and asthma exacerbations (adjusted OR 4.59, 95% CI 1.40–15.07) assessed 10 to 20 years later. This study confirmed the increased SE-IgE positivity in asthmatics and its association to asthma severity and for the first time demonstrated the predictive power of SE-IgE. Being sensitized to SEs—in contrast to inhalant allergens—is associated with an increased risk of subsequently developing severe asthma and asthma exacerbations.

In a Korean study, serum IgE to SEA, SEB, and TSST-1 were detected more frequently in patients with allergic rhinitis and asthma (21–27%) compared to allergic rhinitis patients without asthma (11–21%) and healthy subjects (2–5%) [84]. Similarly, in Japan, 25% of patients with allergic rhinitis were sensitized to SEA/SEB vs. 6.3% of controls [85]. In a meta-analysis of 10 studies, patients with asthma (OR 3.3, 95% CI: 1.6–7.1, *p* = 0.002) and allergic rhinitis (OR 2.4, 95% CI: 1.3–4.7, *p* = 0.008) were significantly more likely than controls to be SE-IgE positive [86]. Studies from Norway [87] have shown an association between *S. aureus* carriage and severe allergic disease as well as allergic multimorbidity. A total of 868 participants of a school-based cohort in late adolescence (aged 18–19 years) showed SE-IgE in serum in 26.2%; SE sensitization, but not *S. aureus* carriage, was associated with poly-sensitization to food and inhalant allergens. SE-sensitized participants also had higher median specific IgE to inhalant allergens, but not to food allergens.

Recently, in the “Learning Early About Peanut Allergy (LEAP)” study, participants’ eczema severity was assessed, skin/nasal swabs were cultured for *S. aureus*, and sensitization to peanut and egg was determined by serum-specific IgE and SPTs [88]. *S. aureus* skin colonization was significantly associated with eczema severity across the LEAP study, whereas at 12 and 60 months of age, it was related to subsequent eczema deterioration. Skin *S. aureus* colonization was further associated with increased levels of egg white and peanut-specific IgE independent of eczema severity. Participants with *S. aureus* were more likely to have persistent egg allergy and peanut allergy at 60 and 72 months of age independent of eczema severity. All but one of the nine participants with a peanut allergy were colonized at least once with *S. aureus*. Thus, *S. aureus* is associated with food sensitization and allergy already early in life.

In summary, several clinical studies show that being sensitized to SEs—in contrast to inhalant allergens—is associated with an increased risk of allergic sensitization and disease severity in patients with chronic inflammatory airway diseases. The molecular mechanisms by which SAgs trigger or amplify allergic immune responses, however, are not yet fully understood.

### 3.2. Interactions of SAgs with Immune Cells

SAgs of *S. aureus* can interact with the human immune system in multiple ways. By activating various immune cell types, they contribute not only to the virulent but also to the allergenic character of this bacterium. The following paragraphs provide an overview on the possible modes of SAg action.

#### 3.2.1. Interaction with T Cells and APCs as SAg and Conventional Antigen

**Figure 2:**
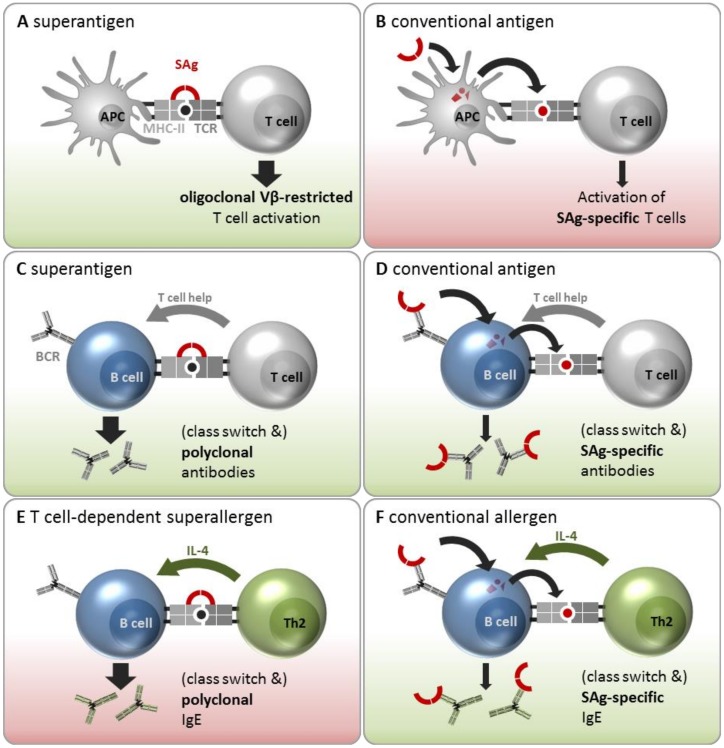
SAgs interact with immune cells in various ways, leading to oligoclonal or antigen-specific activation of T and B cells. (**A**) SAgs cross-link APCs and T cells regardless of the specificity of the TCR, leading to a Vβ-restricted, oligoclonal T cell activation. (**B**) SAgs can be taken up and processed by APCs and subsequently presented as conventional antigens on MHC-II molecules to T cells. T cells whose TCRs are specific for the SAg can bind the MHC:peptide complex and become activated. (**C**) In this special case of SAg-induced immune cell activation, B cells act as APCs. By cross-linking, T cells are activated in a Vβ-restricted manner, as described in A. Moreover, the cross-linked B cells receive activation signals regardless of their B cell receptor (BCR) specificity. This results in the polyclonal B cell activation and the production of polyclonal antibodies. (**D**) SAg-specific B cells bind SAgs via their BCR, followed by processing and subsequent presentation to T cells as conventional antigens on MHC-II molecules. The activated SAg-specific T cells will provide T cell help to the presenting B cells, resulting in B cell activation, plasma cell differentiation, and the release of specific antibodies against the encountered SAg. (**E**) If SAgs cross-link B cells with Th2 cells, the latter are activated in a Vβ-restricted manner and release type 2 cytokines (e.g., interleukin 4 (IL-4)). IL-4 can induce a class switch to immunoglobulin E (IgE) in the cross-linked B cells, resulting in the production of polyclonal IgE. To designate this hitherto unrecognized feature of SAgs, we propose the term ‘T cell-dependent superallergen’. (**F**) SAgs can also act as conventional allergens. In this scenario, SAg-specific B cells bind SAg via their BCR followed by processing and subsequent presentation as conventional antigens on MHC-II molecules to Th2 cells. The activated SAg-specific Th2 cell will provide B cell help and release IL-4, which induces class switch to IgE and the production of specific antibodies against the encountered SAg. Green background color: There is strong scientific evidence for interaction. Red: There is currently only limited evidence for this interaction. Arrows indicate sequence of events.

The term “superantigen” refers to the toxins’ ability to bypass the MHC-II-restricted antigen presentation to T cells by directly cross-linking APCs and T cells independent of the antigen specificity of the T cell (Figure 2A). This does not require SAg uptake and proteolytic processing, but rather depends on its three-dimensional structure. A SAg initially binds to the APC’s MHC-II α- and/or β-chain outside the antigen presentation site and subsequently cross-links it to the TCR on the T cell. In this process, the SAg binds primarily to the TCR’s variable region of the β-chain (Vβ-domain) (or Vα in the case of SEH) [6,32,89]. Each SAg can attach to a characteristic subset of these Vβ elements, which defines its Vβ signature [90]. This Vβ restriction results in an oligoclonal T cell activation [91]. In this manner MHC-II- as well as MHC-I-restricted T cell populations can be targeted, eventually activating up to 20% of T cells [28,92]. Moreover, TSST-1 and SEB contribute to this hyperinduction not only by cross-linking MHC-II molecules and TCRs, but also by stimulating the interaction between CD28 on T cells and its coligand CD86 on APCs. This enhancement was vital for eliciting an inflammatory cytokine response [93,94]. On the APC side, SAgs differ greatly in their binding ability to different MHC-II alleles [95]. SEA was shown to possess two binding sites for MHC-II molecules, enabling attachment to two such molecules on the APC’s surface, which might explain its strong mitogenic potential on T cells [96].

However, SAgs are also conventional antigens (Figure 2B). In fact, many of them are immunodominant, as shown by high specific serum antibody titers in naturally exposed humans [35,36]. In this setting, SAg proteins are taken up by APCs, processed into short peptides, and presented in an MHC-II-restricted manner to T cells. In this case, only SAg-specific Th cells, i.e., those that specifically recognize the SAg peptide in complex with the MHC-II molecule, will get activated. To date, it has not been possible to study SAg-specific Th cells because the strong superantigenic activity of these toxins will overwhelm any antigen-specific response in T cell cultures.However, with the availability of SAg toxoids it will now be possible to enumerate and characterize them.The high frequency of class switched SAg-specific B cells, which is discussed below, strongly implies the existence of SAg-specific T cells, because B cell class switch requires the help of a Th cell recognizing the same antigen.

#### 3.2.2. Interactions with B Cells as SAgs and Conventional Antigens

The conventional antigen-specific T cell-dependent B cell activation, termed ‘cognate interaction’ between the two cell types, requires three signals. The first signal is delivered by the B cell’s binding of an antigen with its specific B cell receptor (BCR). The antigen is then taken up, processed, and peptides are presented on MHC-II molecules to T cells for their activation [97]. The cognate T cell binds with its TCR, upregulates CD40L on its surface, and secretes cytokines [98]. The second and third signals are the ligation of CD40 on the B cell and the binding of cytokines, respectively, which trigger signaling cascades in the B cell leading to its clonal expansion, differentiation, and Ig secretion [99]. Whereas naïve mature B cells express IgD and IgM on their surfaces, T cells help can induce a class switch to IgG, IgA, or IgE. The Ig classes differ in their biological functions, which is important for adapting the humoral immune responses to different antigenic challenges [100,101]. For example, the control of extracellular bacteria or worms requires special immune effector functions that are triggered by specific IgG and IgE, respectively. T cell help via CD40L is required for the Ig class switch in B cells. Finally, T cell help for B cells initiates the affinity maturation of the antibody response, which is caused by somatic hypermutation of the antibody genes [102,103].

SAgs circumvent the antigen-specific interaction between T helper cells and B cells by directly cross-linking MHC-II molecules on B cells with TCRs on T cells (Figure 2C). The T cells would then be activated in a Vβ-specific manner to express CD40L [104] and cytokines which then drive polyclonal B cell activation independent of the antigen-specificities of the T and B cells involved. However, this mechanism of polyclonal B cell activation by SAgs is controversial because experiments have yielded inconsistent results. Several groups have observed inhibition rather than activation of Ig-secreting cells through SAgs in vitro and in vivo, which was T cell-dependent [105,106,107,108]. In contrast, polyclonal B cell activation and differentiation into Ig-secreting plasma cells was observed in cell cultures containing irradiated T cells unable to proliferate. The ratio of B and T cells also influenced the outcome. Cultures with a ratio of 5:1 B/T cells showed highest Ig production, whereas a higher proportion of T cells reduced the Ig production [109]. Other groups found Ig secretion in cultures when they used very low concentrations of SAgs [110,111]. In conclusion, SAgs can be potent polyclonal activators of B cells under conditions that do not elicit strong T cell proliferation. Whether this scenario also takes place in vivo remains to be clarified.

It is clear, however, that SAgs can be recognized by B cells as conventional antigens and induce the production of SAg-specific antibodies (Figure 2D). This implies that SAg-specific B cells bind SAgs via their BCR, internalize and process them, and present the resulting peptides in an MHC-II restricted manner to SAg-specific T cells. These provide cognate T cell help, resulting in B cell activation and the release of class-switched specific anti-SAg antibodies. In fact, class-switched SAg-neutralizing antibodies are highly prevalent in the sera of adults [112]. Moreover, the high specificity of these antibodies for their inducing SAg indicates affinity maturation, which also requires T cell help [35,57,61,63].

#### 3.2.3. Interaction with B Cells as Superallergens and Conventional Allergens

On the basis of epidemiological data (see Section 3.1) and the results of mechanistic studies (see Section 3.2), SAgs are attributed an important role in allergy by initiating or amplifying type 2 responses. Apart from activating Th2 cells in a Vβ-restricted manner (Figure 2A), SAgs might also polyclonally stimulate IgE-positive B cells or even induce a class switch to IgE (Figure 2E). This scenario represents a special case of the SAg-induced polyclonal B cell activation, depicted in Figure 2C. In this—hypothetical—scenario the Th2 cells release IL-4, IL-5, IL-9, and IL-13 [113] and upregulate CD40L upon Vβ-restricted cross-linking of their TCRs with MHC-II molecules on B cells [114]. IL-4 or IL-13 in combination with direct contact to activated T cells via CD40/CD40L drives an Ig class switch to IgE [115]. Thereby, SAgs could initiate or amplify a polyclonal IgE response regardless of the B cells’ antigen specificity.

Several studies corroborate the ability of SAgs to drive polyclonal IgE production. For instance, TSST-1 was shown to induce CD40L expression on oligoclonally activated T cells and to induce IgE synthesis in vitro in a dose-dependent manner. Notably, this IgE synthesis was dependent on direct contact between B and T cells via CD40/CD40L [104]. Furthermore, Hofer and co-workers showed that during the pollen season restimulation of in vivo-primed peripheral blood mononuclear cells with TSST-1 enhanced the allergen-specific IgE production in vitro. This effect was dependent on the ratio of IFNγ and IL-4 in the cell culture. During the pollen season the endogenous IL-4 level was sufficient to induce allergen-specific IgE. In contrast, outside the pollen season the addition of exogenous IL-4 was required to induce IgE secretion due to high endogenous INFγ levels [116]. Moreover, sensitization against SEs was observed to be significantly associated with poly-sensitization against various food and inhalant allergens [87]. We propose the term ‘T cell-dependent superallergen’ to designate this SAg function as a stimulus of B cell poly-sensitization. This concept is distinct from the term “B cell superallergen”, which refers to bacterial or viral proteins that can cross-link conserved structures on IgE thereby triggering the antigen-independent activation and degranulation of mast cells and basophils, which are decorated with IgE via their Igε receptors [117,118].

Finally, the observation of SE-IgE demonstrates that SAgs can also act as conventional allergens (Figure 2F). This implies that they are recognized by specific B cells, taken up, processed, and presented to specific Th2 cells. These provide B cell help and release the pro-allergenic cytokine IL-4 to induce a class switch to IgE, resulting in the production of SE-IgE. Allergen-specific IgE is crucial for specific downstream stimulation of mast cells and basophils causing allergic symptoms.

Recently, Spls were identified as allergens of *S. aureus* [4,70,71]. The immune response against these proteases is characterized by the formation of high titers of specific IgE, release of type 2 cytokines in healthy individuals, and eosinophilic infiltration in the airways upon intratracheal Spl exposure in mice [70]. Similarly, a mutant, non-superantigenic variant of SEC was shown to induce a type 2 cytokine response in mice after subcutaneous injection [55]. Moreover, SAg-specific IgE in patients with atopic dermatitis or airway diseases were observed in different cohorts as described above [72,119,120,121,122,123]. In addition, in nasal polyp mucosal tissue, SEs caused the release of cytokines supporting type 2 inflammation, especially IL-2, IL-4, and IL-5 whereas IL-10 and TGF-β1 were disfavored [124]. Therefore, SAgs appear to have a dual role in allergy, acting on the one hand as T cell-dependent superallergens causing poly-sensitization and on the other hand driving a type 2 immune response as conventional allergens.

#### 3.2.4. Interaction with Other Immune Cells

Staphylococcal SAgs are well-known for their ability to cross-link TCRs and MHC-II molecules and induce oligoclonal T cell activation. However, these molecules can also affect other immune cell types involved in allergy. For instance, SEB can directly cause degranulation of cutaneous mast cells in monkeys [125] and in a rodent RBL-2H3 mast cell line [126]. Similarly, in house musk shrews and marmosets, SEA binds to submucosal mast cells in the gut and triggers degranulation [127,128]. This occurs via an yet-unknown receptor on the mast cells and is independent of MHC-II [30]. When SEB and viable *S. aureus* were added to explanted mucosal tissue from CRS patients, the bacterium was phagocytosed by mast cells and caused mast cell rupture and degranulation. The rate of mast cell degranulation was significantly lower when *S. aureus* was added alone, demonstrating a prominent role for SEB in the uptake of *S. aureus* and mast cell degranulation [129].

Little is known about direct effects of SAgs on eosinophils and basophils. Basophils from AD patients can recognize SAg-IgE complexes via their FcεRI receptor and release histamine [119]. It was also reported that, similar to SpA, SEE has an IgE binding site that targets a conserved framework region of IgE antibodies. Hence, SEE could trigger mast cells and basophils by crosslinking the SEE-specific antigen binding site on one IgE molecule and the conserved framework region on another [130].

## 4. How Does *S. aureus* Benefit from Producing SAgs?

Despite more than 30 years of research, the advantage gained by *S. aureus* from SAgs is still under discussion. SAgs are thought to create an immunological smokescreen through stimulating the release of a storm of cytokines that makes T cells refractory to specific activation. Thus, *S. aureus* can hide from specific immune recognition [9]. Also, T cells exposed to the cytokine storm become anergic and many of them ultimately die [131,132]. Hence, one could speculate that the net effect of SAgs is the reduction of the T cell repertoire including the elimination of *S. aureus*-reactive T cells.

It is also discussed that SAgs promote *S. aureus’* host colonization. SAgs are found in nasal polyp tissues [25,26], their transcripts have been detected in *S. aureus* isolated from the nose of carriers [22], and antibodies directed against SAgs are elevated in persistent carriers [22,29,35]. TSST-1 vaccination reduced the bacterial load in a murine colonization model, presumably by inducing neutralizing antibodies [58]. In another mouse model, in contrast, deletion of SAg genes increased the *S. aureus* burden [133]. To resolve this paradox, it was proposed that SAgs act as a checkpoint for innocuous colonization. By promoting a local inflammatory response that keeps the bacterial density below the pathogenic threshold, SAgs foster asymptomatic carriage, while preventing complete elimination by the immune system [89]. Collectively, these data indicate that SAgs are important for maintaining colonization.

Finally, we have summarized the evidence that SAgs drive a Th2 immune response which can manifest itself in allergy. Driving an allergic inflammation favors *S. aureus* because such a response counteracts the Th1/17 profile, which effectively clears extracellular bacteria. Th1 responses promote macrophage activation and intracellular elimination of phagocytosed bacteria, while Th17 responses initiate neutrophil maturation and recruitment, and they enhance epithelial and mucosal barrier functions [50]. Thus we have proposed that type 2 immunity, which can be promoted by SAgs, represents an immune evasion mechanism [4,134]. It is, therefore, not surprising to notice that the rate of colonization of *S. aureus* among allergic patients, such as atopic dermatitis and CRS, is significantly higher than in healthy individuals [4,135,136]. It is conceivable that type 2 immune modulation by *S. aureus* also increases the risk of infections by this microorganism as well as by other bacteria or enhances their severity [137].

Overall, it seems like *S. aureus* has evolved a plethora of SAgs with multiple functions: (1) create a “smokescreen” that prevents mounting a specific T cell response against these bacteria, (2) foster colonization, and (3) promote an allergenic environment within the host that increases the survival chances of the bacterium.

## 5. Open Questions

The data compiled in this review show that there is growing evidence for a role of SAg in inducing and amplifying allergic responses, and possible mechanisms, both evidence-based and hypothetical, are provided. However, this review also pinpoints open questions that should be addressed in the future:

- Currently, specific IgE antibodies can be measured against four different SAgs, SEA–SEC, and TSST-1, using commercial tests. To evaluate the potential roles of the other 22 SAgs in allergic diseases the appropriate assays need to be established.
- SAg-specific T cells, i.e., those that specifically recognize a SAg peptide in complex with a MHC-II molecule, have not been studied to date. To understand their role in allergic diseases, however, it is crucial to determine the quality of the natural T cell response against SAgs in healthy individuals and patients. With the availability of SAg toxoids and sophisticated T cell assays, this will now be feasible.
- T cell-dependent superallergens are thought to stimulate B cells of any specificity to form IgE. However, evidence to support this notion of SAg-mediated polysensitization is scarce.
- Considering the high prevalence of anti-SAg antibodies, including SE-IgE, it is likely that most individuals are primed with *S. aureus* SAgs and have generated an anti-SAg T cell response, which may comprise substantial numbers of Th2 cells. Vaccination with SAg toxoids without adjuvant might enhance this pre-existing Th2 bias in susceptible individuals, thereby exacerbating allergy rather than inducing protection. To avoid this, the SAg-specific T cells responses have to be studied in healthy individuals and patient cohorts.
- After more than 30 years of research, the evolutionary advantages gained from SAgs are still under discussion. Using SAg-knock-out strains or SAg vaccines in animal models could help to scrutinize their role in colonization and infection.
- Since SAgs likely play an important role in allergic disorders, future research should enforce efforts to develop broadly cross-reactive SAg vaccines or SAg antagonists.

## 6. Closing Remarks

There is strong epidemiological evidence for an involvement of *S. aureus* SAgs in allergic diseases. Patients with airway diseases show significantly higher rates of *S. aureus* colonization than healthy individuals. Moreover, SE-IgE can be found in a relevant subgroup of patients suffering from upper and lower type 2 airway diseases. Notably, only sensitization to SEs but not inhalant allergens is associated with an increased risk of developing severe asthma and asthma exacerbations in the future. However, the molecular mechanisms by which SAgs trigger or amplify allergic immune responses are not yet fully understood. SAgs could have a dual role in allergies, acting on the one hand as T cell-dependent superallergens causing B cell poly-sensitization, and on the other hand driving a type 2 immune response as conventional allergens. In addition, SAgs could directly trigger mast cell degranulation and hence exacerbate allergic symptoms. Future research should address the IgE response against all known *S. aureus* SAgs, decipher the natural T cell response to SAgs as conventional antigens as well as their potential role as superallergens. A better understanding of the role of SAgs in atopy might guide the development of novel diagnostic and therapeutic tools for upper and lower type 2 airway diseases.

## Figures and Tables

**Table 1:** Clinical trials involving active vaccination with SAg toxoids or passive vaccination with therapeutic antisera.

Vaccination Type	Target	Name (Company; NCT Number ^1^)	Study Design	Status and Study Results	Intervention	Duration	Ref.
Active	SEB ^2^	STEBVax (Integrated BioTherapeu- tics; NCT00974935)	Non-randomized, dose escalation	Phase I, completed. STEBvax was safe, well-tolerated and immunogenic, induced/boosted toxin-neutralizing antibodies	STEBVax vaccine ^3^ with Alhydrogel adjuvant, six doses (10 ng–20 µg) or 20 µg given in two vaccinations 21 days apart	02/11- 03/15	[48]
Active	TSST-1 ^4^	rTSST-1v ^5^ (Biomedizi- nische Forschungs GmbH; NCT02340338)	Randomized, double-blind, adjuvant- controlled dose escalation	Phase I, completed. rTSST-1v was safe, well-tolerated, and immunogenic, induced/boosted toxin-neutralizing antibodies	rTSST-1 variant ^5^ with Al(OH)_3_, six doses in one to two vaccinations (100 ng–30 µg)	06/14- 06/15	[49]
Active	TSST-1	rTSST-1v (Biomedizi- nische Forschungs GmbH; NCT02814708)	Randomized, placebo- controlled	Phase II, ongoing	rTSST-1 variant ^3^ with Al(OH)_3_, two doses (10, 100 µg) in one to three vaccinations	Since 03/16	-
Passive	SAgs	IVIG ^6^ (Hospices Civils de Lyon; NCT02219165)	Randomized, placebo- controlled	Phase II, completed	IVIG (single dose, 2 g/kg)	Since 01/15	-

^1^ ClinicalTrials.gov Identifier (https://clinicaltrials.gov/), ^2^ SEB, staphylococcal enterotoxin B, ^3^ STEBVax is a recombinant detoxified version of the SAg SEB lacking toxic and superantigenic properties. The mutant contains mutations in the hydrophobic binding loop (L45R), the polar binding pocket (Y89A), and the disulfide loop (Y94A), thereby disrupting the interaction of the toxin with human MHC-II molecules. ^4^ TSST-1, toxic shock syndrome toxin 1, ^5^ TSST-1variant (TSST-1v) is a recombinant detoxified version of the SAg TSST-1 lacking toxic and superantigenic properties. The double mutation G31R-H135A impairs rTSST-1v binding to both MHC-II molecules and to the TCR. ^6^ IVIG, intravenous immunoglobulins.
